# The bispecific antibody targeting VISTA and PD-L1 shows enhanced tumor inhibitory activity in pancreatic, endometrial and breast cancers compared to mono- and combination immune checkpoint blockade

**DOI:** 10.3389/fimmu.2025.1486799

**Published:** 2025-05-09

**Authors:** Przemysław Bielski, Jan Barczyński, Michał Mikitiuk, Maja Myrcha, Kamil Rykała, Louis Boon, Wiktoria Gąsior, Aleksandra Hec-Gałązka, Tad A. Holak, Tomasz Sitar

**Affiliations:** ^1^ Recepton Sp. z o.o., Gdansk, Poland; ^2^ Department of Organic Chemistry, Faculty of Chemistry, Jagiellonian University, Krakow, Poland; ^3^ JJP Biologics Sp. z o.o., Warsaw, Poland

**Keywords:** VISTA, PD-L1, PD-1, bispecific, antibodies, combination therapy, cancer

## Abstract

**Background:**

The introduction of checkpoint immunotherapeutic agents in the last decade has revolutionized cancer treatment. Although anti-PD-1, anti-PD-L1 and anti-CTLA4 are promising therapies, many patients fail to respond or relapse due to drug resistance potentially due to redundancy of immune checkpoints. One of the ways to improve the efficacy of this cancer treatment is to target two or even three immune checkpoints. To date, the benefit of combined anti-VISTA/anti-PD-L1 therapy has been confirmed, but no one has investigated the efficacy of blocking these negative immune checkpoints with a bispecific anti-VISTA/anti-PD-L1 antibody.

**Methods:**

In this study, the bispecific antibodies (bsAbs) were produced in three formats: symmetric (IgG-HC-scFv), asymmetric (Fab-scFv-Fc(KIH)) and 2 x scFv. The binding and blocking properties of these bispecific antibodies (bsAbs) and their efficacy compared to monotherapy and combination therapy were then determined using endometrial (RL95-2), pancreatic (PANC-1) and breast (BT-20) cancer cell lines.

**Results:**

The bsAbs generated in this study showed weaker binding properties to PD-1 and VISTA in ELISA (EC_50_) than the parent antibodies (atezolizumab and onvatilimab). Blockade of VISTA/VSIG-3 binding was also weaker with bsAbs compared to onvatilimab, but the ability to block the PD-1/PD-L1 pathway was slightly better than with atezolizumab. The Fc-based bsAbs showed statistically significant higher levels of lysis of endometrial, breast and pancreatic cancer cells. The symmetric bsAbs (IgG-HC-scFv) showed the most promising therapeutic potential. Higher levels of cancer cell lysis were associated with higher levels of pro-inflammatory cytokines. Both the asymmetric and symmetric bsAbs resulted in higher secretion levels of IFN-γ, TNFα and Granzyme B than anti-VISTA, anti-PD-L1 monotherapy and anti-VISTA/anti-PD-L1 combination therapy.

**Conclusion:**

The high level of tumor cell lysis and increased expression of pro-inflammatory cytokines induced by the Fc-based bsAbs suggest a novel approach for the treatment of pancreatic, endometrial and breast cancer.

## Introduction

In the recent decade, immune checkpoint inhibitors (ICIs) have been increasingly incorporated into the treatment of various cancers and have become a standard part of oncology treatment protocols (1–3). However, a significant proportion of cancer patients still exhibit poor responses and significant toxicity profiles to ICI therapy (4, 5). This trend highlights the need for further research and development of personalized cancer treatment strategies to improve outcomes for this subset of patients. One approach to improving cancer treatment is combination therapy involving two or more immune checkpoints.

ICI therapy is currently based on monoclonal antibodies that block cytotoxic T-lymphocyte antigen 4 (CTLA-4), programmed cell death 1 (PD-1), and programmed cell death ligand 1 (PD-L1) (1–3). A relatively new and promising negative immune checkpoint is the V-type immunoglobulin suppressor of T-cell activation (VISTA) (6). As shown by Kakavand et al. (7) in metastatic melanoma and Gao et al. (8) in prostate cancer, expression of VISTA increased after anti-PD-1 or anti-CTLA-4 treatment. The percentages of VISTA and PD-L1 expression on tumor-infiltrating CD4+ and CD8+ T cells and CD68+ macrophages were significantly increased after therapy, suggesting that both checkpoint proteins may contribute to the immunosuppressive tumor microenvironment (TME) and resistance to negative immune checkpoint blockade therapy (8).

Tumor expression of VISTA increases after anti-PD-1 or anti-CTLA-4 treatment (7, 8); for example, 43% of patients treated with anti-PD-1 monotherapy relapsed within 3 years due to acquired resistance with high VISTA tumor expression (9). In glioma patients, VISTA tumor expression was higher in stage III and IV than in stage I and II, and high VISTA and PD-1 mRNA correlated with poor overall survival in glioma patients (10). In mouse models of cancer, anti-VISTA (13F3) antibodies significantly inhibited cancer growth (11). Thus, anti-VISTA - anti-PD-L1 bsAbs may be a promising strategy to overcome resistance to current immune checkpoint therapies. In principle, the bsAbs offer several advantages compared to monoclonal antibodies or their combination: (a) Immune cell recruitment and superior cytotoxicity; (b) bsAbs can block proteins from multiple pathways simultaneously; (c) bsAbs can penetrate tissues that are inaccessible to monospecific antibodies (12, 13); (d) bsAbs form immunological synapses and activate immune cells: (e.g. AFM13) (13); (e) bsAbs facilitate receptor clustering, which can lead to increased target internalization and degradation (14); (f) bsAbs might engage tumor cells with immune cells.

Here, we present results that suggest a new direction in immunotherapy using bsAbs anti-VISTA/anti-PD-L1.

## Materials and methods

### Materials

All antibodies: anti-PD-L1 (atezolizumab, Ate), anti-VISTA (onvatilimab, Onv), the asymmetric bispecific antibodies anti-PD-L1/anti-VISTA (bsAb-1), the symmetric bispecific antibodies anti-PD-L1/anti-VISTA (bsAb-2), anti-VISTA/anti-PD-L1 in 2 x single-chain variable fragment format (2 x scFv) (bsAb-3), and all proteins: VISTA (R1_008), VSIG-3 (R1_038), PD-1 (R1_001), PD-L1 (R1_002) were produced by Recepton. Genetic constructs were synthesized by Eurofins Genomics (Ebersberg, Germany). Plasmid DNA midiprep (cat. K210004) was purchased from Thermo Fisher Scientific (Waltham, MA, USA). All restriction enzymes and T4 ligase (cat. M0202L) were purchased from New England Biolabs (Ipswich, MA, USA). Culture media for CHO (cat. 94120) and HEK (cat. 9413) were purchased from Fujifilm Irvine Scientific (Santa Ana, CA, USA). L-glutamine (cat. HN08.3) was purchased from Carl Roth (Karlsruhe, Germany). RPMI-1640 (cat. 30-2001), Dulbecco’s Modified Eagle’s Medium (DMEM) (cat. 30-2002), DMEM: F-12 (cat. 30-2006) and Eagle’s Minimum Essential Medium (EMEM) (cat. 30-2003) were purchased from ATCC (Manassas, VA, USA). Trypsin (cat. 25200-072) was purchased from Thermo Fisher Scientific. Fetal bovine serum (cat. P30-19375), hygromycin B (cat. P06-08100) and DPBS (cat. P04-361000) were purchased from PAN Biotech (Aidenbach, Germany). G418 (geneticin, cat. G073-39US) was purchased from TOKU-E (Bellingham, WA, USA). Polyethylenimine linear (25 kDa) (cat. 23966-100) was purchased from Polysciences (Warrington, PA, USA). 10x Phosphate-Buffered Saline was made with 80 g NaCl (cat. 27810.295, VWR (Radnor, PA, USA)); 2.0 g of KCl (cat. 0395, VWR); 14.4 g of Na2HPO4 (cat. 117992300, Chempur (Piekary Śląskie, Poland)); 2.4 g of KH2PO4 (cat. 26925.295, VWR) and pH adjusted to 7.4. It was then diluted 1:10 to achieve 1× working solution. PBST was made by adding 0.5% Tween 20 (cat. M147, VWR) to the 1× PBS. PD-1/PD-L1 blockade bioassay (cat. J1250) was purchased from Promega (Madison, WI, USA).

### Design of antibody constructs

Atezolizumab and onvatilimab were designed based on the sequences found on DrugBank (52CMI0WC3Y) and NIH (1UI8F5IIZ4), respectively. bsAb-1 (asymmetric antibody) is a bispecific antibody designed in a knob-into-hole IgG1 format targeting both PD-L1 and VISTA. It consists of two halves: the first half consists of a light chain (LC) and a heavy chain (HC) derived from Ate, while the second half contains a single-chain variable fragment (scFv) derived from Onv. The scFv is fused to the N-terminus of the HC derived from Ate. To facilitate bispecificity, specific amino acid mutations (Y349C, T366S, L368A, and Y407V) were introduced on one heavy chain of the symmetric IgG1 Fc structure within the anti-PD-L1 variable region. This mutation creates a structural “groove” or hole that serves as a binding site for the bispecific antibodies. In addition, amino acid mutations (S354C and T366W) have been introduced into the second heavy chain of the IgG1 Fc structure within the fused anti-VISTA scFv. The scFv consists of the variable regions of the light and heavy chains joined by a (GGGGS)4 linker. bsAb-2 (symmetric antibody) is another bispecific antibody, but in an IgG4 format that lacks the knob-into-hole mutation. This antibody targets PD-L1 and VISTA and consists of a light chain (LC) and the variable region of the heavy chain derived from Ate. However, the heavy chain has been changed from IgG1 to IgG4. The scFv targeting VISTA is attached to the C-terminus of the CH3 domain by a (GGGGS)2 linker. bsAb-3 is a third format of the tested antibody anti-VISTA/anti-PD-L1, it is in 2 x single-chain variable fragments format (BiTe). This bsAb was designed as follows: VISTA (VL)-(GGGGS)4-VISTA(VH)-(GGGGS)4-PD-L1 (VH)-(GGGGS)4-PD-L1(VL). Immune checkpoints were constructed from sequences found in UniProt. Immune checkpoint receptors and antibody constructs are listed in **Supplementary Table S1**.

### Generation of stable cell lines

Immune checkpoints and antibodies were generated in a stable CHOK1 cell line. Genetic constructs were cloned into expression plasmids, linearized, and cells were transfected by electroporation, followed by selection with increasing concentrations of G418 (geneticin) (TOKU-E). Productivity of each generated cell line was assessed by high performance liquid chromatography (HPLC), cells with satisfactory productivity were propagated and 1 L batch of each protein was produced.

### Protein purification

All cultures were centrifuged on the day of harvest and the supernatant was filtered through a 0.2 µm polyethersulfone filter (Advanced Microdevices, Ambala Cantt, India). The purification procedure for Ate included affinity chromatography with protein A, virus inactivation, anion exchange chromatography, cation exchange chromatography and size exclusion chromatography. The purification strategy for Onv and bsAb-2 consisted of affinity chromatography with protein A and size exclusion methods. bsAb-1 was purified by affinity chromatography, cation exchange chromatography and size exclusion. bsAb-3 purification strategy consisted of affinity chromatography with Ni-Sepharose and size exclusion methods. Immune checkpoint purification procedure included: Protein A affinity chromatography and size exclusion chromatography. All chromatography steps were performed on AKTA Pure 25 and are described below. Protein A affinity chromatography was used as the first purification step. A column packed with MabSelectSure (Cytiva) was prepared and washed with 4 column volumes (CV) of 20 mM NaHPO4 + 150 mM NaCl at pH 7.0. Protein samples were applied to the column with a contact time of 4 minutes. The columns were then washed with 3 CV of NaHPO4 + 150 mM NaCl pH 7.0 and 4 CV of 40 mM NaAc pH 5.5. The proteins were then eluted with 40 mM NaAc pH 3.3 and subjected to virus inactivation by adjusting the pH to 3.6 with 1 M TRIS pH 8.0 followed by incubation for 1 hour. Proteins were then neutralized to pH 6.8 with 1 M TRIS pH 8.0 and filtered.

Affinity chromatography on Ni-Sepharose Excel was used as the first purification step for 2 x scFv. A column packed with Ni-Sepharose Excel (Cytiva) was equilibrated with 4 column volumes (CV) of 20 mM NaHPO4 + 250 mM NaCl at pH 7.4. Protein samples were applied to the column with a contact time of 4 minutes. The columns were then washed with 3 CV of 20 mM NaHPO4 + 250 mM NaCl at pH 7.0. Proteins were then eluted with 20 mM NaHPO4 + 250 mM NaCl + 300 mM imidazole at pH 7.0. Flow-through anion-exchange chromatography was used to remove host cell proteins and DNA that could lead to immunogenicity, antibody degradation or aggregation. A 1 mL Sartobind Q column was sanitized with 1 M NaOH, regenerated with 1 M NaCl, and equilibrated with 40 mM NaAc + 10 mM Tris pH 7.4, maintaining a conductivity of less than 5 mS/cm. The protein load was adjusted to pH 7.4 and loaded onto the membrane. The column was washed with 40 mM NaAc + 10 mM TRIS and the flow was collected to 30 mAU. For the purification of monoclonal antibodies, cation exchange chromatography (CEX) is used to separate proteins with different charge variants. In the case of bsAb purification, the presence of multiple domains and flexible linkers can introduce structural heterogeneity leading to the capture of different conformational states. This can result in the observation of multiple peaks during the CEX purification step. Praesto SP 45 resin equilibrated with 40 mM NaAc + 10 mM NaCl pH 5.5 was used. The protein sample was adjusted to pH 5.5 and applied to the column. The column was washed with the same buffer and elution was performed using a salt gradient. The eluted protein was neutralized to pH 6.8. Size exclusion chromatography (SEC) was used as the final purification step for all antibodies. A Superdex 200 pg 26x600 column (Cytiva) equilibrated with phosphate-buffered saline was used to separate aggregates or “incomplete” proteins. Protein samples were adjusted to pH 7.4 and loaded onto the column. Separation was performed continuously at a flow rate of 1 mL/min.

Immune checkpoints and antibodies concentrations were measured after purification using the Tecan SPARK plate reader at 280 nm.

### ELISA

ELISA was used to determine the affinity of antibodies to receptors: ELISA plates (Nunc MaxiSorp, Thermo Fisher) were coated with 50 μL solution of antibodies at the 5 µg/mL concentration and left overnight in a refrigerator (4°C). The next day, the plates were equilibrated at room temperature (RT) (1h), washed (4 x 300 µL PBST), and blocked (1 hour, RT, without shaking) with 1% BSA solution (Fisher Scientific). PD-L1 or Vista were diluted to the concentration range 50-0,0003 µg/mL in PBS, added to the appropriate wells (50 µL) and incubated for 1 hour (RT, with shaking). The plates were then washed (4 x 300 µL PBST) and anti-His-tag antibodies were added at a dilution of 1:10000 (cat. 4603-08, SouthernBiotech) and incubated for 1 hour (RT, without shaking). After incubation, the plates were washed again (4 x 300 µL PBST) and secondary antibodies (Strep-HRP, cat. 21124, Thermo Fisher) were added at 1:10000 dilution (50 µL) - 1 hour incubation (RT, without shaking). Finally, the plates were washed (6 x 300 µL PBST) and 100 µL prewarmed TMB solution (Sigma-Aldrich) was added to each well. The assay was developed for 6 minutes followed by the addition of stop solution (0.2 M H_2_SO_4_). Absorbance (450 nm and 655 nm for background subtraction) was read on a Tecan Spark microplate reader, and data were analyzed using GraphPad Prism software.

Competitive ELISA: ELISA plates (Nunc MaxiSorp, Thermo Fisher) were coated with 50 μL solution of PD-L1 or Vista at the 10 µg/mL concentration and left overnight in a refrigerator (4°C). The next day, the plates were equilibrated at RT, washed (4 x 300 µL PBST) and blocked (1 hour, RT, without shaking) with 1% BSA solution (Fisher Scientific). The tested antibodies were diluted to the concentration range 20-0,01 µg/mL in a solution of biotinylated ligands at constant concentration (VSIG – 5 µg/mL; PD-1 – 15 µg/mL), added to the corresponding wells (50 µL) and incubated for 1 hour (RT, with shaking). The plates were then washed (4 x 300 µL PBST) and primary antibodies (Strep-HRP, cat. 21124, Thermo Fisher) were added at 1:10000 dilution (50 µL) - 1 hour incubation (RT, without shaking). Finally, the plates were washed (6 x 300 µL PBST) and 100 µL prewarmed TMB solution (Sigma-Aldrich) was added to each well. The assay was developed for 6 minutes followed by the addition of stop solution (0.2 M H2SO4). Absorbance (450 nm and 655 nm for background subtraction) was read on a Tecan Spark microplate reader, and data were analyzed using GraphPad Prism software.

### PD-1/PD-L1 blockade bioassay

The PD-1/PD-L1 immune checkpoint bioassay (PD-1/PD-L1 Bioassay, Promega) was performed according to the manufacturer’s instructions. PD-L1+ aAPC/CHO-K1 cells were plated at 40x10^4^ cells in 100 µL medium (Ham’s F12, 10% FBS) in 96-well white flat-bottomed assay plates and incubated overnight at 37°C, 5% CO2. The next day, the medium was removed from the assay plate and serially diluted antibodies were added at 40 µL per well in assay buffer (RPMI1640 + 1% Fetal Bovine Serum (FBS)). PD-1 effector Jurkat cells were then resuspended in assay buffer (RPMI 1640 + 1% FBS) at a concentration of 1.25 x 10^6^ cells/mL and added to the assay plate at 40 µL per well (total of 50 x 10^4^ cells). Cells were cocultured for 6 hours (37°C, 5% CO2), then removed from the incubator and equilibrated at room temperature for 5 minutes. Bio-GloTM Reagent (Promega) was prepared according to the manufacturer’s instructions and added to each well at 80 µL per well. Assay plates were incubated at RT for 15 minutes and luminescence was measured using a Tecan Spark microplate reader. Data were analyzed using GraphPad Prism software.

### Human tumor cells killing assay

Peripheral blood mononuclear cells (PBMCs) were isolated by Ficoll gradient centrifugation (Ficoll Paque Plus, Cytiva) from human blood samples (obtained from the Regional Center for Blood Donation and Treatment in Gdansk, Poland). After isolation, the cells were cryopreserved in 90% FBS (FBS Good, PANBiotech), 10% DMSO (Sigma Aldrich). One day before the experiment, human tumor cells RL95-2 (ATCC), BT20 (ATCC) and Panc 1 (ATCC) were plated on 96-well plates (3x10^4^ cells/well) in appropriate growth medium. Simultaneously, PBMC effectors were thawed and left overnight in RPMI 1640 medium (ATCC), 10% FBS (FBS Good, PANBiotech) and 50 IU/mL IL-2 (Sigma-Aldrich). Compounds were tested at 10 nM concentration while maintaining an effector:target (E:T) cell ratio of 10:1. Assays were incubated at 37°C with 5% CO2 for 120 hours. Plates were then washed with DPBS to remove PBMC and 20 µL MTT (5 mg/mL, PanReac AppliChem) was added to each well and incubated for 2 hours, followed by the addition of 100 µL MTT Crystal Dissolver (10% SDS, 0.01 N HCl). The plates were left in an incubator (37°C, 5% CO2) overnight. The next day, absorbance was read at 570 nm with background subtraction at 690 nm (Tecan Spark). The percentage of cell lysis was calculated as the ratio of compound treated samples (human tumor cells + PBMC + tested compounds) to PBMC treated samples (human tumor cells + PBMC). Statistical analysis was performed using GraphPad Prism software.

### Cytokine release assay

Samples for the cytokine release study were collected after 120 hours of PBMC/cancer cell line coculture at 37°C. Analysis was performed according to the following protocol. A 96-well microplate was coated with 100 µL of capture antibody (IL-2 and TNF-α - 4 µg/mL; IL-10 and INF-γ - 2 µg/mL; Granzyme B - 800 ng/mL) and incubated overnight at RT. Each well was aspirated and washed three times with wash buffer (1xPBS + 0.05% Tween 20). Plates were blocked by adding 300 μL of Reagent Diluent (1% BSA in PBS, pH 7.2-7.4) to each well. The plate was incubated at RT for 1 hour. The washing step was repeated. 100 μL of samples or standards in Reagent Diluent were added per well. The plate was covered with an adhesive strip and incubated for 2 hours at RT. The washing step was repeated. 100 μL detection antibody diluted in Reagent Diluent was added to each well. The plate was covered with a new adhesive strip and incubated for 2 hours at RT. The washing step was repeated. 100 µL streptavidin-HRP was added to each well. The plate was covered and incubated for 20 minutes at RT. The wash step was repeated. 100 µL TMB (Sigma Aldrich) was added to each well. The wells were incubated at room temperature for 20 minutes. 100 µL Stop Solution was added to each well. The plate was gently vortexed. Optical density was determined using a microplate reader set at 450 nm (655 nm background subtraction).

### Leukocytes phenotyping and killing effect of PBMCs on tumor cells assay

To verify the changes in lymphocytes phenotypes two multiparameter panels were used. The first contained anti-CD45-PerCP-Cy5.5 (BD Biosciences, cat. 564105), anti-CD11b-PE (BD Biosciences, cat. 555388), anti-CD14-APC (BD Biosciences, cat. 555399), anti-CD15-PE-Cy7 (BD Biosciences, cat.560827) and anti-HLA-DR-FITC (BD Biosciences, cat. 555811). The second one was consisted of anti-CD45-PerCP-Cy5.5 (BD Biosciences, cat. 564105), anti-CD3-APC-H7 (BD Biosciences, cat. 560176), anti-CD4-PE-Cy7 (BD Biosciences, cat. 557852), anti-CD8-FITC (BD Biosciences, cat. 570817), anti-CD25-APC (BD Biosciences, cat. 340907) and after fixation/permabilization procedure anti-FoxP3-PE (BD Biosciences, cat. 560046). Settings adjustment and gating was performed on stained, untreated PBMC’s, separately for both panels.

Co-culture of RL95-2 and PBMC cells were conducted on 6-well plates for 120hours with E:T (effector to target) ratio of 10:1. Bispecific and monospecific antibodies were add in concentrations of 10 nM, while combination of monospecific antibodies in concentration of 10 + 10 nM. At the end of the incubation, PBMC’s were harvested and centrifuged (5 minutes, 200g). For each sample, 1x10^6^ cells were resuspended in 100 µl of flow cytometry staining buffer (BD Pharmingen™ Stain Buffer (FBS), cat. 554656) and stained according to the manufacturer’s instructions (separately for two panels, without FoxP3 staining). Samples were incubated on ice for 30 minutes, and next washed two times with 2mL of flow cytometry staining buffer (5 minutes, 200g). After last wash, samples for the first panel were resuspended in 300 µL of staining buffer and analyzed, while samples for the second panel were fixated/permeabilized (Invitrogen, eBioscience™ Foxp3/Transcription Factor Staining Buffer Set, cat. 00-5523-00). Shortly, after last wash cell pellets were resuspended in 1mL of 1X Foxp3 Fixation/Permeabilization Solution and pulse vortex, followed by 30 minutes incubation (RT, protected from light). Next, cells were washed two times with 1X Permeabilization Buffer (5 minutes, 500g). After last wash, cells were resuspended in 100 µL of 1X Permeabilization Buffer and anti-FoxP3 antibody was add, followed by 30 minutes incubation (RT, protected form light). Again, cells were washed two times with 1X Permeabilization Buffer (5 minutes, 500g) and finally resuspended in 300 µL of staining buffer and analyzed. All analysis were performed on BD Lyric cytometer and BD FACSutie software (ver. 1.6.0.3066). RL95-2 cell lysis percentage (%) determined according to the “Human tumor cell killing assay” procedure.

### Statistical analyses

Statistical analyses were performed using GraphPad statistical software (GraphPad Software Inc). All experiments were repeated at least three times under identical experimental conditions. EC50 values were calculated using nonlinear regression analysis. Statistical significance of tumor cell lysis and cytokine concentration between groups was determined by one-way ANOVA. Student’s t-test was used to compare the statistical significance of changes in the percentage of cytokine concentration in the culture medium after 72 and 120 hours of incubation. Data were considered statistically significant when p values were less than 0.05 (*p <0.05, **p <0.01, and ***p<0.001).

## Results

### Structure, purity and binding characterization of bispecific anti-PD-L1/anti-VISTA antibodies

BsAbs can be designed as a Fc-based symmetric and asymmetric formats, as well as in an Fc-free bsAbs (2xscFv). The relatively large molecular weight of the Fc-based bsAbs helps to purify and improve solubility and stability, increase the serum half-life and affinity, and thereby enhance biological activity (15, 16). Fc-free bsAbs have shorter half-lives but demonstrates improved tissue distribution, penetration and clearance properties. The use of single-chain antibody fragments (2xscFvs) is considered as safer than full-length antibodies. Bispecific T cell Engager (BiTe) is one of the most widely used formats of 2xscFvs (17).

**Figure 1:**
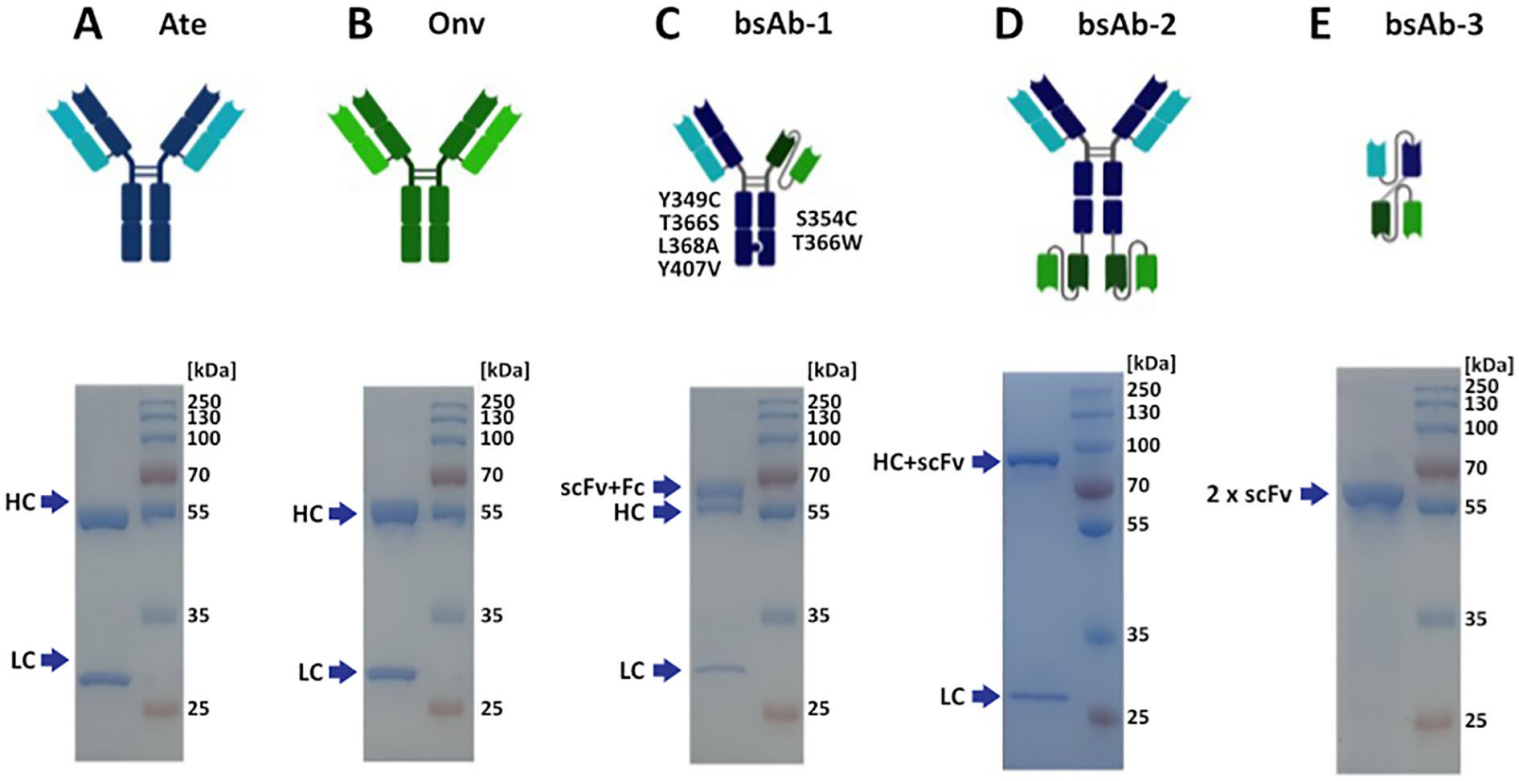
Structures and SDS-PAGE profiles of the antibodies. The quality of the antibodies was determined on 12.0% acrylamide gels. Protein bands were visualized with Coomassie Brilliant Blue R presented by SDS-GEL: **(A)** atezolizumab, antibody anti-PD-L1, Ate; **(B)** onvatilimab, antibody anti-VISTA, Onv; **(C)** asymmetric bsAb anti-PD-L1/anti-VISTA, bsAb-1; **(D)** symmetric bsAb anti-PD-L1/anti-VISTA, bsAb-2; **(E)** 2 x scFv anti-PD-L1/anti-VISTA, bsAb-3. HC - heavy chain (CH3, CH2, CH1, VH); LC - (CL, VL); Fc - crystallizable fragment (CH3, CH2); scFv - single chain fragment variable (VL and VH).

The biological activity of the obtained antibodies was confirmed by ELISA analysis. BsAbs in symmetric, asymmetric and 2 x scFv formats were constructed. **Figure 1** shows schematic representation of the parental and bispecific antibodies. Monospecific antibodies migrate in two bands under reducing conditions (**Figures 1A, B**), originating from a heavy chain (HC) (~50 kDa), which migrates a few kDa higher due to glycosylation, and a light chain (LC) (~25 kDa). LC of symmetric and asymmetric antibody migrated as typical LC (**Figures 1C, D**). HC of bsAb-1 showed 2 bands (**Figure 1C**). The “higher” band corresponds to the arm comprising CH3, CH2 of atezolizumab and scFv of onvatilimab. The “lower” band corresponds to the arm containing CH3, CH2, CH1 and VH of atezolizumab. HC of the symmetric antibody (bsAb-2) migrated around 90 kDa (**Figure 1D**). HC of bsAb-2 migrated higher than the theoretical molecular weight (MW) due to additional linkers connecting the scFv of Onv to the Fc of R1_A6. The MW is higher than the theoretical molecular weight, which can be caused by glycosylation and the presence of the linker. The linker can increase the shape of the molecule. bsAb-3 is a bsAb containing 4 variable fragments connected by linkers. As expected, the antibody migrates as a single band at 65 kDa (**Figure 1E**). The theoretical MW is 56 kDa; an additional 10 kDa is derived from the 3 linkers present in the construct. All antibody solutions were >95% pure, as determined by SDS-PAGE.

**Figure 2:**
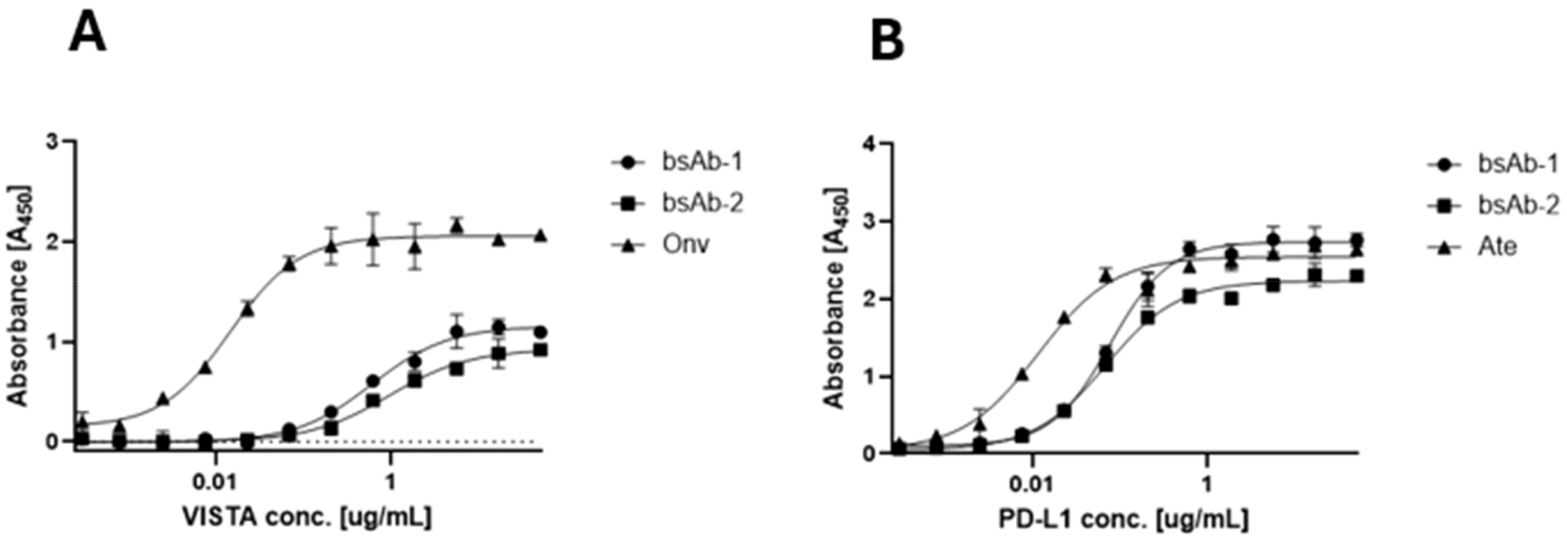
Comparison of binding activity of monospecific (Ate and Onv) and bispecific antibodies (bsAb-1 and bsAb-2) by ELISA. **(A)** Comparison of binding affinity of onvatilimab (Onv) and bispecific antibodies (asymmetric - bsAb-1 and symmetric - bsAb-2) to VISTA; **(B)** Comparison of binding affinity of atezolizumab (Ate) and bispecific antibodies (asymmetric - bsAb-1 and symmetric - bsAb-2) to PD-L1. ELISA plates were coated with 0.5 ng VISTA **(A)** or PD-L1 **(B)**. Then 2.5-0.00015 ng of Vista **(A)** or PD-L1 **(B)** was added.

The affinities of the antibodies to human PD-L1 and VISTA were evaluated by ELISA. As shown in **Figure 2A**, the binding affinity of bsAb-1 and bsAb-2 to VISTA was 4.52 nM and 4.59 nM, respectively. These bsAbs showed weaker binding than onvatilimab Onv (0.1 pM). With respect to PD-L1 (**Figure 2B**), the affinity of bsAb-1 and bsAb-2 was 0.58 nM and 0.33 nM, respectively, both less potent than atezolizumab (0.07 nM) as shown in **Figure 2**. bsAb-3 was not tested but demonstrated the ability to inhibit the interaction between PD-1/PD-L1 and VISTA/VSIG-3 as shown in the competitive ELISA assay.

### Blocking properties of the bispecific antibodies

**Figure 3:**
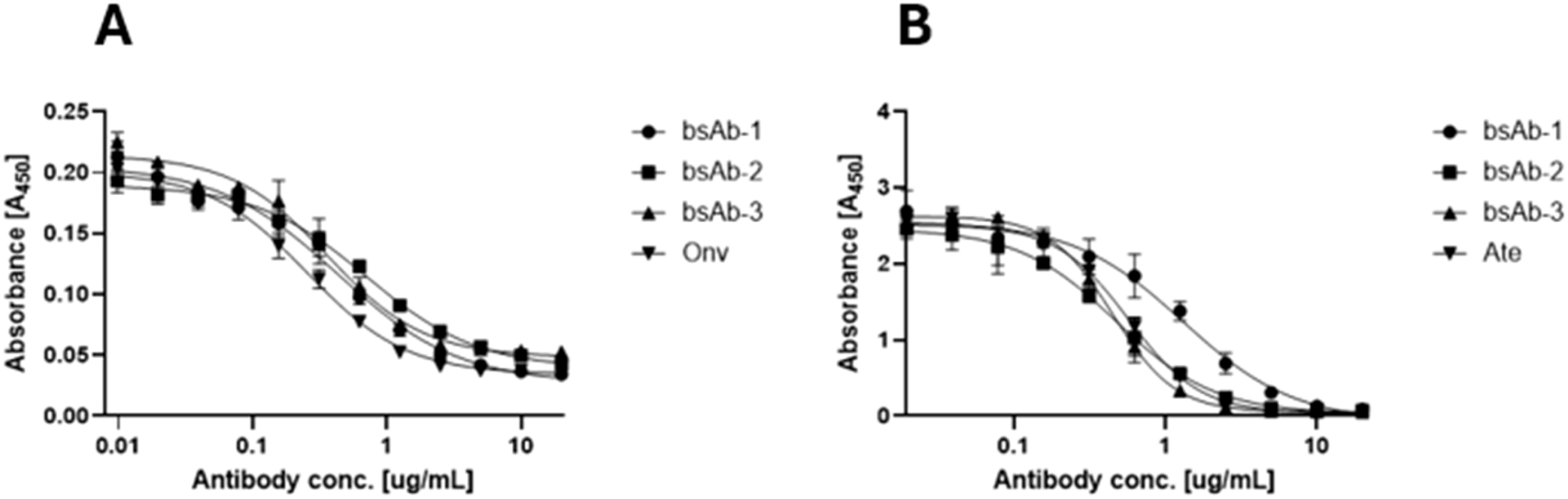
BsAbs block the interaction between VISTA/VSIG-3, PD-1/PD-L1 by ELISA. Competitive ELISA assay showing the blocking activity of Onv, bsAb-1, bsAb-2 and bsAb-3 on the interaction of VISTA/VSIG-3 **(A)** and the blocking activity of Ate, bsAb-1, bsAb-2 and bsAb-3 on the interaction of PD-1/PD-L1 **(B)**. ELISA plates were coated with 0.5 ng VISTA **(A)** or PD-L1 **(B)**. Then 0.25-0.0005 ng of antibody was added in a solution containing 0.25 ng biotinylated VSIG **(A)** or 0.75 ng biotinylated PD-1 **(B)**.

**Figure 4:**
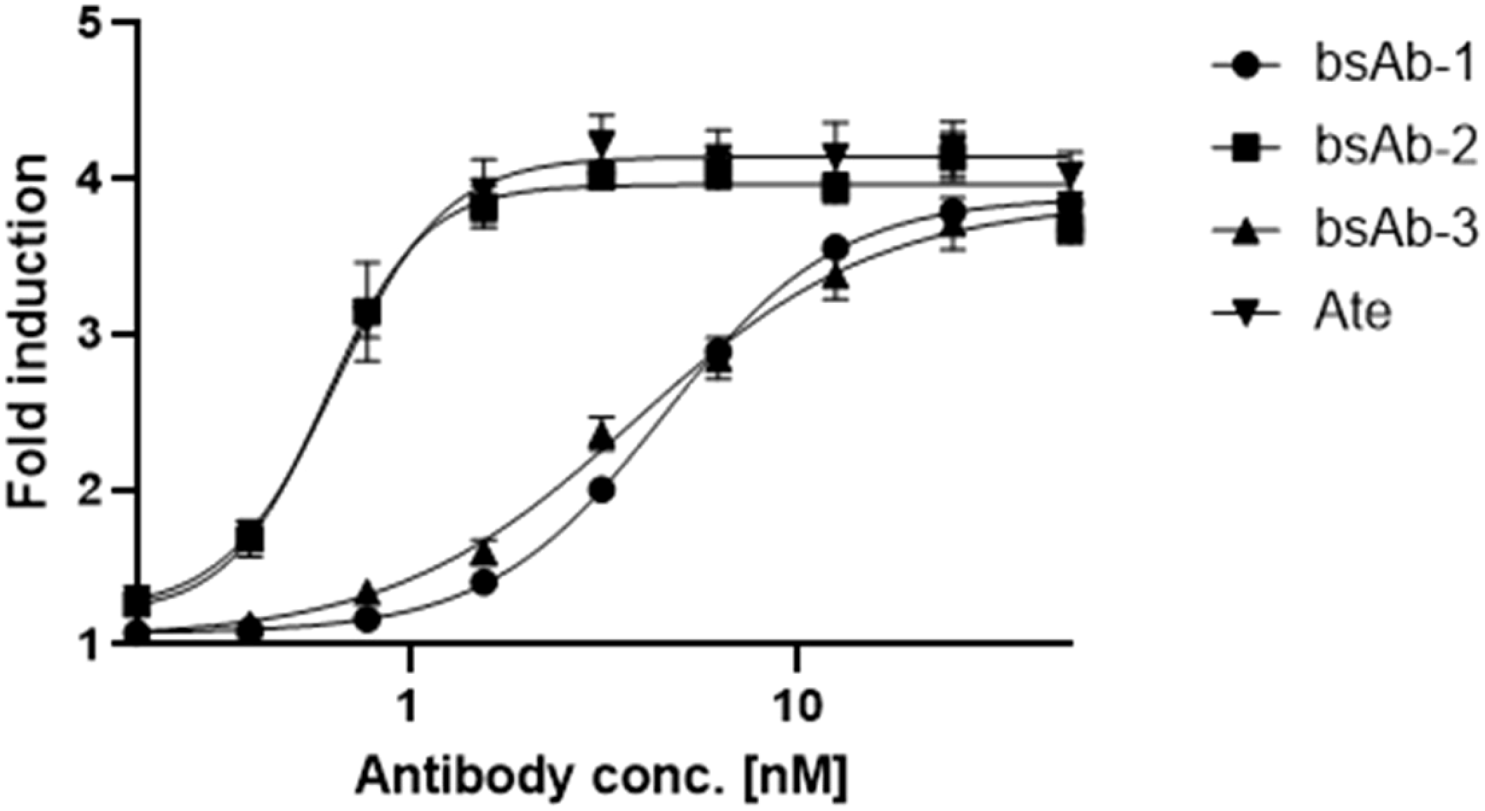
*In vitro* biological activity of bsAbs. The blocking activity of Ate, bsAb-1, bsAb-2 and bsAb-3 to block the PD-1/PD-L1 interaction studied using the PD-1/PD-L1 bioassay. PD-L1+ aAPC/CHO-K1 cells were plated at 40x104 cells in 100 µL medium (Ham’s F12, 10% FBS) in a 96-well plate. The next day, were added 50-0,20 nM antibodies with 50 x 104 cells per well of PD-1 effector Jurkat cells at 40 µL per well.

The bsAbs were designed to restore the function of non-responsive T cells for cancer treatment by blocking PD-L1 and VISTA immune checkpoints on cancer and immune cells. Competitive ELISA was used to verify the blocking effect of the antibodies on PD-1/PD-L1 and VISTA/VSIG-3 *in vitro*. The results confirmed that all bsAbs were able to block the interaction of PD-1/PD-L1 and VISTA/VSIG-3. As presented in **Figure 3A**, asymmetric bsAb (EC_50_ 3.28 nM), symmetric bsAb (EC_50_ 3.41 nM) and 2 x scFv (EC_50_ 6.5 nM) blocked VISTA/VSIG-3 interaction weaker than onvatilimab (Onv) (EC_50_ 1.72 nM). In **Figure 3B**, bsAb-2 (EC_50_ 2.27 nM) ability to block PD-1/PD-L1 pathway was slightly better than atezolizumab (EC_50_ 3.94 nM). bsAbs-1 (EC_50_ 9.92 nM), similar to 2 x scFv (EC_50_ 8.04 nM), had weaker blocking properties than the parental antibody (Ate). In addition, the PD-1/PD-L1 blocking bioassay confirmed the ability of Ate and bsAbs to block the pathway in a cellular system as shown in **Figure 4**. The EC_50_ for bsAb-2 (0.61 nM) was similar to that of atezolizumab (0.65 nM). The EC_50_ for bsAb-1 was 4.52 nM and was close to the EC_50_ for 2 x scFv (3.81 nM).

### Effect of the antibodies on lysis of RL95-2, BT-20 and Panc-1 cancer cells in the PBMC assay

After evaluating the potential to block immune receptor-ligand interactions, the next and critical *in vitro* test was to evaluate the efficacy of the bsAbs in a peripheral blood mononuclear cell (PBMC) cytotoxicity assay.

**Figure 5:**
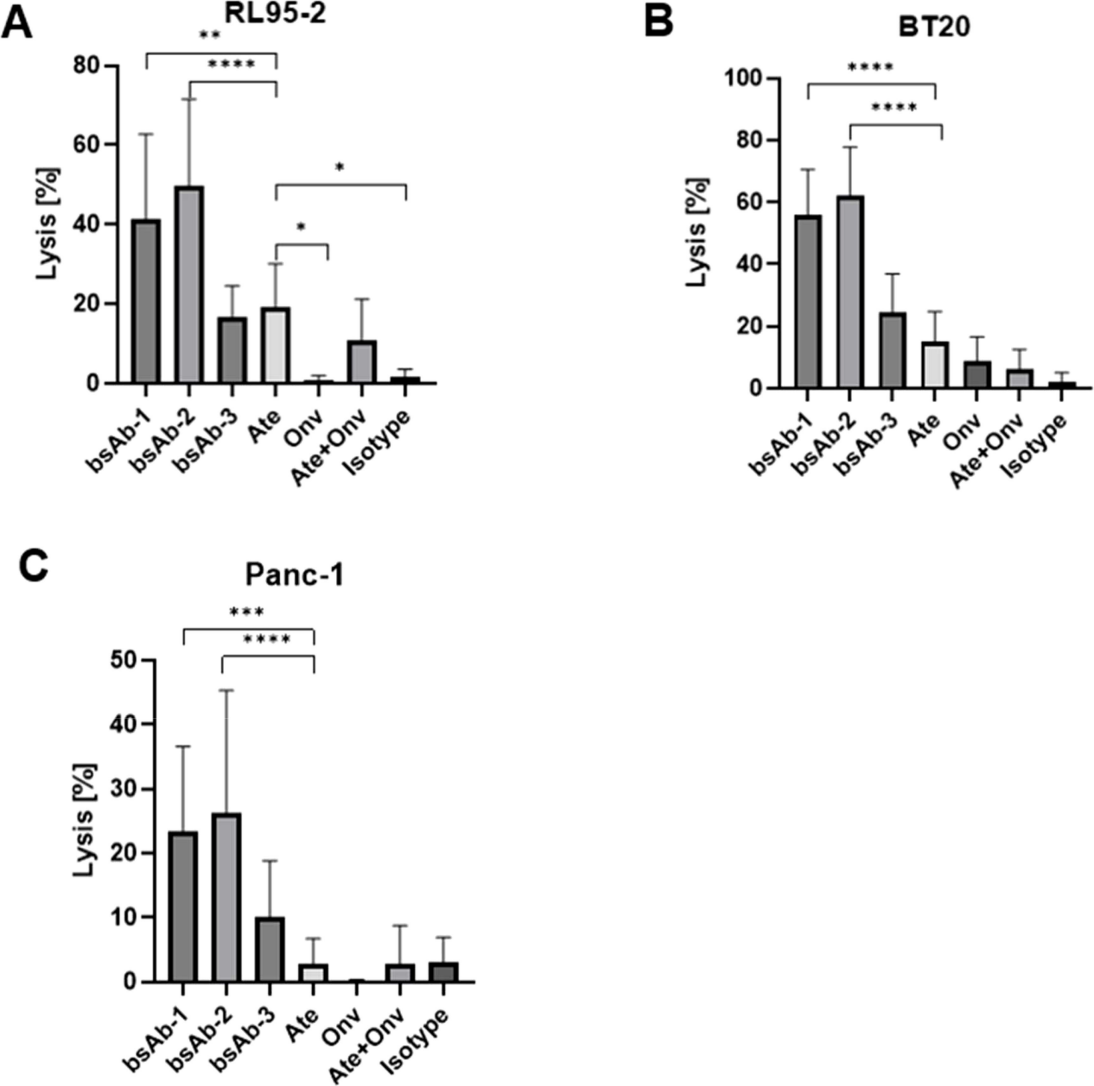
Antibodies induce PBMCs potential to kill RL95-2, BT20 and Panc-1 cells. The cells lysis values **(A–C)** for RL95-2, BT20, and Panc-1, respectively. Data are derived from human PBMCs from 4 healthy donors. All experiments were repeated three times. Data were considered statistically significant when p values were lower than 0.05 (*p <0.05, **p < 0.01, ***p<0.001 and ****p <0.0001) (Onv: anti-VISTA, Ate: anti-PD-L1, bsAb-1: asymmetric bsAb, bsAb-2: symmetric bsAb, bsAb-3: 2xscFv, A6+A2: Onv + Ate, Isotype: ipilimumab).

The effect of the antibodies on the lysis of the RL95-2 cells is shown in **Figure 5A**. Mulati et al. (18) and Gabr and Gambhir (19) found high expression of VISTA in endometrial cancer. Based on these articles, the RL95-2 cell line was selected for the assay. The combined therapy (anti-PD-L1 + anti-VISTA) (10.76%) showed a significantly higher lysis of cancer cells compared to the control group (p < 0.05). Surprisingly, however, the combination was not more effective than the use of atezolizumab alone (Ate) (p = 0.07). All bsAbs were significantly more effective than control and anti-VISTA monotherapy (p < 0.05). Among the bsAbs, the 2 x scFv format showed the lowest efficacy (16.85%), which was inferior to both anti-PD-L1 monotherapy (p = 0.58) and combination therapy (p = 0.12). Fc-based bsAbs (bsAb-1 - 41.41%) (bsAb-2 - 49.55%) showed significantly higher cytotoxicity than all other groups (p < 0.05). bsAb-2 was the most effective, most likely due to the valency of these antibodies. Similarly, Santich found that tetravalent antibodies were more effective than bivalent antibodies (20).

The second type of cell line tested was the breast cancer line (BT-20). The effect of the antibodies on the lysis of BT-20 cells is shown in **Figure 5B**. The monotherapy anti-PD-L1 (15.02%) showed significantly higher cytotoxicity against cancer cells compared to the control group (2.14%) (p < 0.05) and the combination therapy anti-PD-L1 + anti-VISTA (6.38%) (p < 0.05). Similar to Ate, anti-VISTA monotherapy was significantly better than control (p < 0.05). Monotherapy anti-PD-L1 was slightly better than monotherapy anti-VISTA (8.68%), however not significant (p = 0.09). All bsAbs showed a higher degree of lysis than the other groups. Monotherapy anti-PD-L1 showed significantly worse efficacy than bsAb-1 (55.99%), bsAb-2 (62.06%) and bsAb-3 (24.67%) (p < 0.05). 2 x scFv showed a lower degree of cancer cell lysis than Fc-based bsAbs (p<0.05). The highest degree of lysis was observed for bsAb-2, but the result was not significantly better than bsAb-1 (p = 0.34).

Finally, **Figure 5C** shows the effect of the antibodies on the lysis of PANC-1 cells. The last cell line was selected based on results reported by Hou et al. (21), which showed that VISTA is highly expressed in 25.6% of tumor cells (TCs), 38.1% of immune cells and 26.0% of endothelial cells in 223 pancreatic ductal adenocarcinoma (PDAC) tumor tissues. bsAb-2 showed the highest level of cancer cell lysis (26.17%) and was similar to bsAb-1 (23.31%) (p = 0.69). Fc-based bsAbs induced a significantly higher level of cancer cell lysis than bsAb-3 (10.03%), Ate (2.88%), Onv (0.08%), Onv + Ate (2.79%) and the isotype control group (2.96%) (p < 0.05). bsAb-3 induced a significantly weaker cytotoxic process than Fc-based bsAbs, but significantly better than monotherapy, combination therapy and control group (p < 0.05). Anti-PD-L1 monotherapy and anti-PD-L1 + anti-VISTA combination therapy (p = 0.96) showed similar levels of cytotoxicity against cancer cells compared to the control group (p = 0.96 and p = 0.94, respectively). Anti-VISTA monotherapy was significantly weaker than all other groups (p < 0.05) except combination therapy (p = 0.16). This result is surprising, but the values for control, combination and monotherapy are less than 3%, so the difference may be due to method variability. Antibodies induce PBMCs potential to kill RL95-2, PANC-1 and BT20 cells. Lysis scores along with calculated p-values are shown in the **Supplementary Material** (**Supplementary Figure S1**).

### Effect of the antibodies on cytokine release

**Figure 6:**
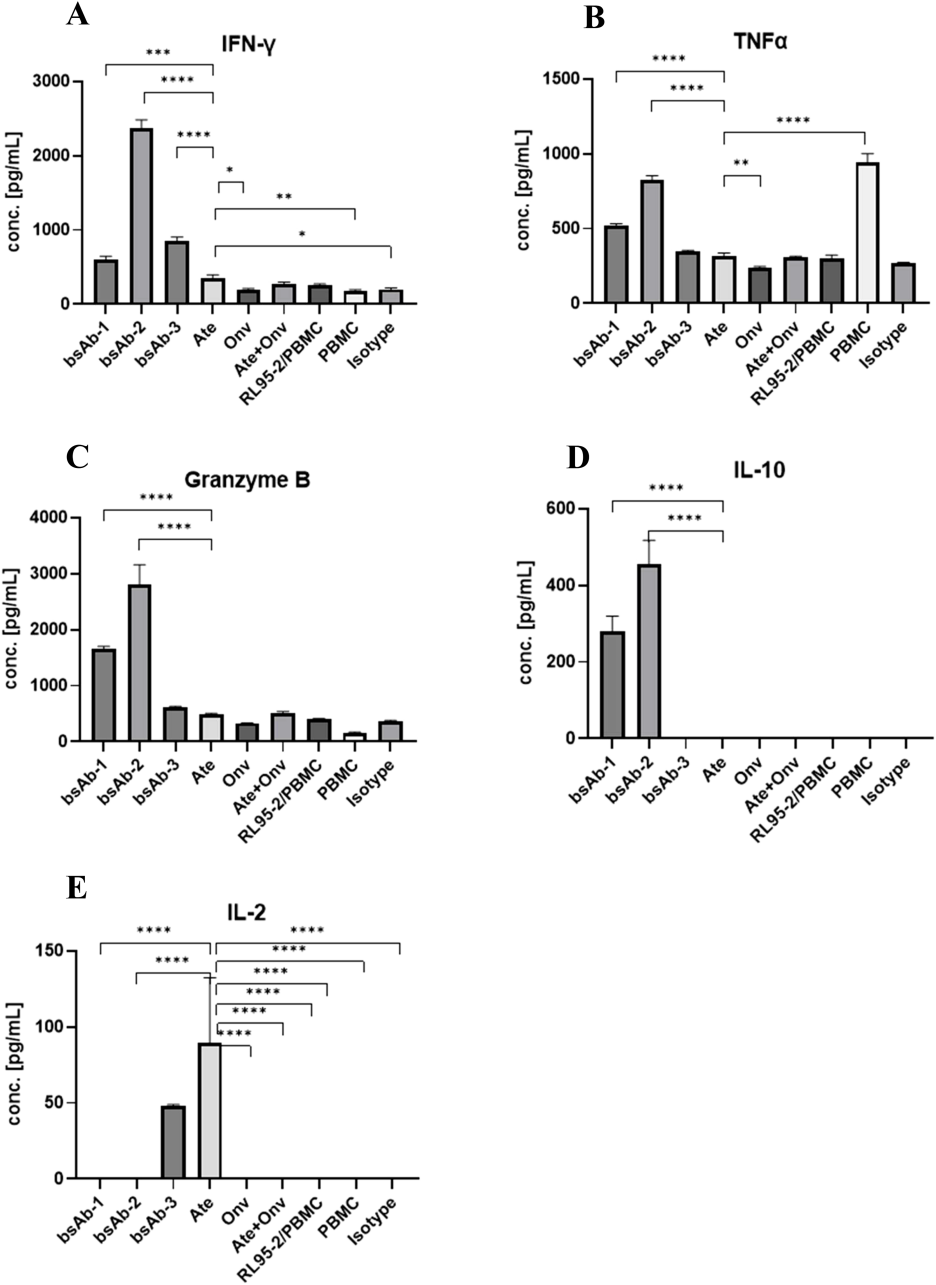
Antibodies induce cytokine release by PBMCs co-cultured with RL95. The INF-γ, TNFα, Granzyme B, IL-10 and IL-2 concentration (**A–E**, respectively). Data are derived from human PBMCs from 1 healthy donor. Each value is an average from 3 independent samples. Data was considered statistically significant when p values lower than 0.05 (*p <0.05, **p < 0.01, ***p<0.001 and ****p <0.0001) (Onv: anti-VISTA, Ate: anti-PD-L1, bsAb-1: asymmetric bsAb, bsAb-2: symmetric bsAb, bsAb-3: 2xscFv, A6+A2: Onv + Ate, Isotype: ipilimumab).

We tested the effect of the bsAbs and antibodies on IL-2 release (**Figure 6E**). A high level of IL-2 was observed for atezolizumab and 2 x scFv. The level of IL-2 was almost 2 times higher for PBMC treated with atezolizumab (89.61 pg/ml) than for PBMC treated with bsAb-3 (48.16 pg/ml), but the difference was not statistically significant (p > 0.17). In the other samples including both the symmetric and asymmetric bsAbs, the concentration of IL-2 was below the detection limit.

For IL-10 release (**Figure 6D**), the level of IL-10 was high in both the symmetric bsAb-2 (455.0 pg/mL) and asymmetric bsAb-1 (281.2 pg/mL), while it was below the detection limit in the other conditions tested. The level of interleukin in bsAb-1 was almost 1.5 times lower than in bsAb-2 and it was statistically significant (p < 0.05).

The effect of the antibodies on Granzyme B (**Figure 6C**) release was as follows: Granzyme B for Fc-based bsAbs (bsAb-2 (2807 pg/ml) and bsAb-1 (1650 pg/ml)) was significantly higher than in the sample with bsAb-3 (622.7 pg/ml), ATE (486.4 pg/mL), Onv (331.2 pg/mL), Onv+Ate (519.3 pg/mL), RL95-2+PBMC (402 pg/mL), PBMC (154.1 pg/mL) and isotype control (353.9 pg/mL) (p < 0.05). Symmetric bsAb (bsAb-2) induced a statistically significant higher level of Granzyme B than asymmetric bsAb (bsAb-1) (p < 0.05). In the sample treated with bsAb-3 and Onv + Ate, the level of the cytokine was significantly higher than in the sample with PBMC only (p < 0.05). Granzyme B levels were not significantly different between the other samples (p > 0.05). Statistical analysis between all treatments is shown in **Supplementary Figure S2**.

In terms of IFN-γ release (**Figure 6A**), the level of IFN-γ was significantly higher for bsAbs than for samples from other groups (p < 0.05). By far the highest concentration of the cytokine was measured for bsA-2 (2377 pg/ml) and was significantly higher (p < 0.05) than in the sample treated with bsAb-1 (596.1 pg/ml) and bsAb-3 (847.8 pg/ml). We did not observe any dependency between Fc fragment and IFN-γ concentration, in contrast to Granzyme B and IL-10. Fc-free bsAb induced significantly higher cytokine levels than asymmetric bsAb (p < 0.05). Among the remaining conditions, the highest concentration of IFN-γ was measured in the atezolizumab-treated sample (352.4 pg/mL) however this is about 7 fold lower than with the symmetric bsAb-2. Statistical analysis between all treatments is shown in **Supplementary Figure S2**.

Finally, for TNFα release (**Figure 6B**): The highest level of TNFα was measured in the untreated sample (PBMC only) (945 pg/ml) and it was significantly higher than for other treatments (p < 0.05). Again, the cytokine level for Fc-based bsAbs (bsAb-1: 521 pg/ml, bsAb-2: 828.7 pg/ml) was significantly higher (p < 0.05) than in samples treated with bsAb-3 (349. 9 pg/ml), Ate (318.6 pg/ml), Onv (235.1 pg/ml), Ate + Onv (307.5 pg/ml), controls (PBMC + RL95-2: (300.1 pg/ml) and isotype control (268.7 pg/ml). Statistical analysis between all treatments is shown in **Supplementary Figure S2**.

### Leukocytes phenotyping and killing effect of PBMCs on tumor cells assay

**Figure 7:**
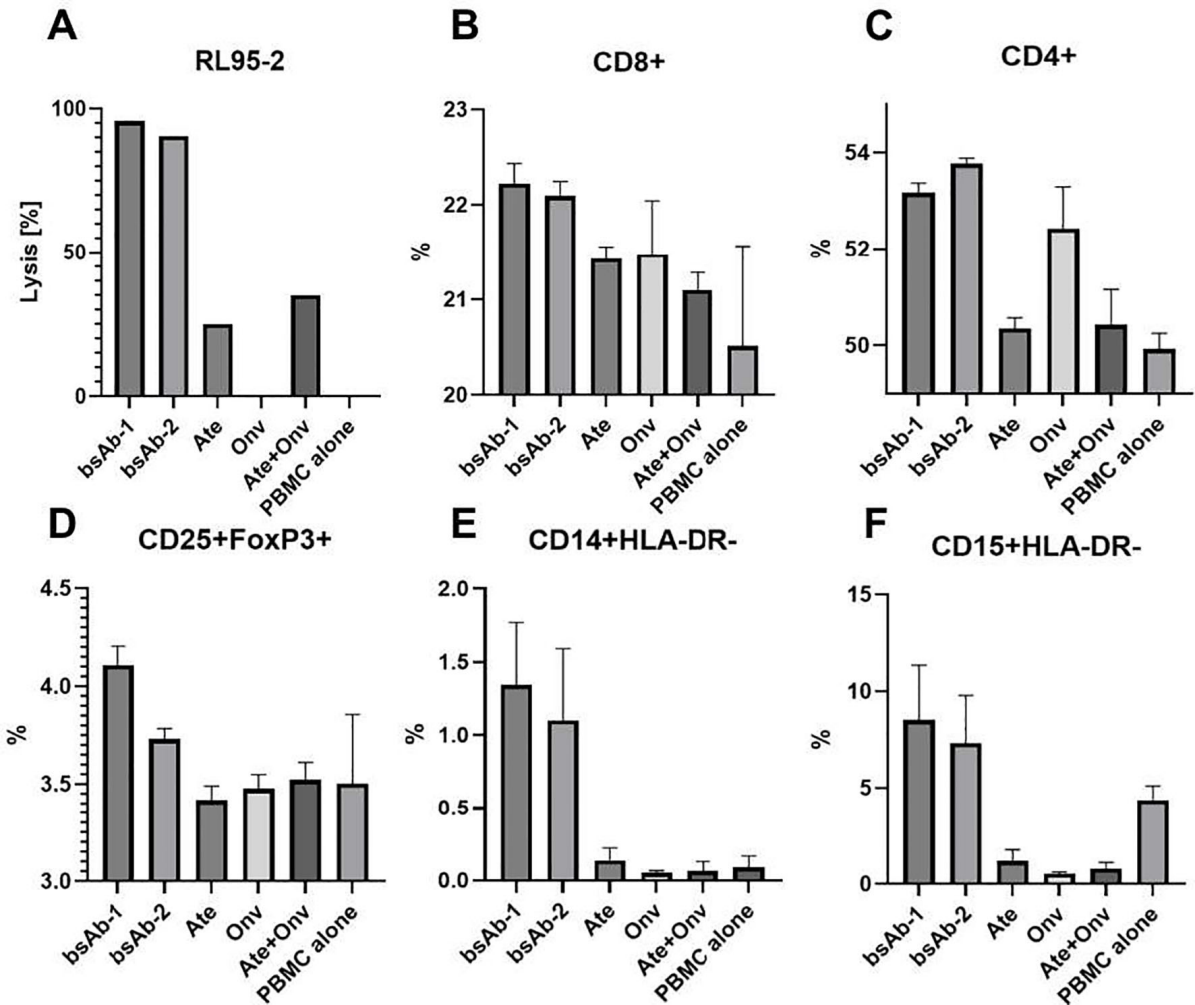
Leukocytes phenotyping and killing effect of PBMCs on tumor cells assay. **(A)** RL95-2 cells lysis level. The CD4+, CD8+, CD25+FoxP+, CD14+HLA-DR- and CD15+HLA-DR- levels (**B-F**, respectively). Gating path: CD4+ (CD45+>CD3+>CD4+), CD8+ (CD45+>CD3+>CD8+), CD25+FoxP3+ (CD45+>CD3+>CD4+>CD25+>FoxP3+), CD14+HLA-DR- (CD45+>CD11b+>CD14+>HLA-DR-), CD15+HLA-DR- (CD45+> CD11b+> CD15+>HLA-DR-). Data are derived from human PBMCs from 1 healthy donor. All experiments for leukocytes phenotyping were repeated three times. Data was considered statistically significant when p values lower than 0.05 (Onv: anti-VISTA, Ate: anti-PD-L1, bsAb-1: asymmetric bsAb, bsAb-2: symmetric bsAb, bsAb-3: 2xscFv, A6+A2: Onv + Ate, PBMC alone: PBMC).

To gain a more precise understanding of the increased tumor cell lysis under the influence of bsAbs, we conducted an analysis of changes in lymphocyte populations and myeloid-derived suppressor cells (**Figures 7B–F**). Based on the literature (18, 19), the RL95-2 cell line was selected for the study.

**Figure 7A** presents the level of tumor cell lysis in the cell phenotyping experiment. Statistical significance was not marked on the graph because, due to the low number of cells, the cell lysis test was conducted in a single repetition to confirm that bsAbs induce a higher level of cytotoxicity (**Figure 7A**). This result suggests that the primary level of cytotoxicity arises from PD-L1 blockade, while additional VISTA blockade enhances this effect, which is supported by others (22, 23).

As shown in **Figure 7B**, bsAbs (bsAb-1 – 22,23%, bsAb-2 – 22,10%) induced a slightly higher level of CD8+ lymphocytes compared to other groups (Ate – 21,43%; Onv – 21,48%; Ate+Onv – 21,10%; PBMC alone – 20,51%) and statistical significance was observed only in comparison to the control group (PBMC alone) (p<0.05). No statistically significant differences were observed between the remaining groups.

As seen in **Figure 7C**, bsAbs and the monospecific anti-VISTA antibody induced an increase in helper lymphocytes (CD4+). The level of CD4+ lymphocytes under the influence of bsAb-1 (53,18%), bsAb-2 (53,78%), and Onv (52,44%) was statistically significantly higher than in the other groups (Ate – 50,35%; Ate+Onv – 50,43%, PBMC alone – 49,93%) (p<0.05).

Only the bsAb-1 (4,80%) resulted in the highest levels of regulatory T lymphocytes (CD25+Foxp3+, Tregs) (**Figure 7D**). Only under this condition the level of Tregs was significantly higher than in the other groups (bsAb-2 – 4,62%; Ate – 3,55%; Onv – 3,49%; Ate+Onv – 3,6%; PBMC alone – 3,39%). The bsAb in a symmetric format did not cause a significantly higher level of CD25+Foxp3+ lymphocytes but still resulted in higher levels than in the monospecific antibody groups. Similar correlations were observed in the study by Le Mercier et al. (11).

The increased levels of myeloid-derived suppressor cells (MDSCs) remain a topic of discussion. BsAbs led to the highest levels of MDSCs. Both M-MDSCs (CD15+HLA-DR-) (bsAb-1 – 1,34%; bsAb-2 – 1,10%; Ate – 0,14%; Onv – 0,05%; Ate+Onv – 0,06%; PBMC alone – 0,09%) (**Figure 7F**) and PMN-MDSCs (CD14+HLA-DR-) (bsAb-1 – 8,52%; bsAb-2 – 7,33%; Ate – 1,19%; Onv – 0,53%; Ate+Onv – 0,80%; PBMC alone – 4,38%) (**Figure 7E**) were significantly higher in than in the other groups. In the literature, MDSC levels in the tumor immune microenvironment (TIM) decrease (11, 24), which may be due to the fact that our study was conducted *in vitro*. Statistical analysis between all treatments is shown in **Supplementary Figure S3**.

## Discussion

The main goal of this project was to block two immunosuppressive pathways simultaneously with a single bsAb. PD-L1 is present on cancer cells and various types of immune cells including APC and naïve CD4 T cells and its presence directly reduces the cytotoxic effect of T lymphocytes. Like PD-L1, VISTA is also present on cancer cells, but also on monocytes and antigen-presenting cells (APCs), leading to suppression of the immune system. The use of the bsAb against these two targets is expected to induce a higher immune response compared to the corresponding combination therapy through trans interactions between immune cells (25, 26). BsAbs generally have acceptable side effects; for example, preclinical studies have shown that ABL503 (PD-1 × 4-1BB) is well tolerated with a low risk of liver toxicity and superior activity compared to a combination of the corresponding monoclonal antibodies (27). In addition, bsAbs are expected to have a reduced ability to cause resistance. The anti-LAG-3 × anti-PD-L1 bsAb caused a decrease in LAG-3 expression, whereas the use of monoclonal antibodies led to an increase in LAG-3 expression (28). In the present study, we observe a higher level of tumor cell lysis as a result of the use of bsAbs compared to monospecific antibodies and the combination of anti-VISTA + anti-PD-L1. This result suggests a promising positive outcome in immunotherapy. Interestingly alongside, with the higher level of tumor cell lysis, we also observed a high level of pro-inflammatory cytokines.

Clinical trials of onvatilimab, a parental anti-VISTA antibody, were discontinued due to cytokine release syndrome (29). One solution to this problem is the development of pH-dependent antibodies (e.g. SNS-101, (30)), which are only active at pH below 7. Another solution could be the amino acid substitutions (e.g., L234F, L235E, and N297G) in the Fc region, creating tailor-made Fc-silent mutations that prevent non-specific immune cell activation (27). Another approach is to change the antibody format from IgG1 to IgG4, which reduces the ADCC effect. In our study, we tested bsAbs in two formats: an asymmetric IgG1 format antibody and a symmetric IgG4 format antibody. Both Fc-based bsAbs showed increased cytokine levels compared to the other groups. The absence of lower cytokine levels with the IgG4 format may be due to the greater efficacy of the bsAb in the symmetric format due to its higher valency compared to the asymmetric antibody. The 2 x scFv format seems promising since tumor cell lysis levels with the 2 x scFv were higher than with monotherapy and the anti-VISTA/anti-PD-L1 combination. Cytokine levels with 2 x scFv were similar to control and to atezolizumab.

An additional feature of VISTA compared to PD-L1 is its high expression on MDSCs, which indirectly reduce T lymphocyte activity. In the TME, MDSCs accumulate to suppress immune function and promote tumor growth through the induction of tumor-derived factors, cytokines and/or chemokines (31). As presented by Mortezaee and Majidpoor (32), blocking these two suppressive immune checkpoints on myeloid cells enhances immune system activation. This may explain the high level of tumor cell lysis observed in our study. As shown in **Figure 7**, there was an increase in the levels of cytotoxic T cells (CD8+), helper T cells (CD4+), Tregs (CD25+Foxp3+) and MDSCs following the application of bispecific anti-VISTA/anti-PD-L1 antibodies. The increase in CD8+ and CD4+ cells was expected and consistent with the literature. Surprisingly, higher levels of CD25+Foxp3+ and MDSC cells were also observed. Xu et al. (24) and Le Mercier et al. (11) observed decreased levels of these cells in the TIM. Differences in our study may be due to the fact that our research was conducted *in vitro*. On the other hand, the results obtained highlight the importance of MDSCs in the search for anti-cancer therapies.

In conclusion, the studies presented here show that the use of bsAb targeting VISTA and PD-L1 antibodies results in a higher level of tumor cell lysis in pancreatic, breast and endometrial cancers than monospecific antibodies and their combination. The 2 x bispecific scFv format bsAbs deserve special attention as they did not cause an increase in cytokine levels. A potential risk of using Fc-dependent anti-VISTA/anti-PD-L1 bsAbs is the occurrence of cytokine storm. Therefore, modifications that reduce Fc-dependent cytotoxicity may be further investigated and tested in future animal models.

## Data Availability

The original contributions presented in the study are included in the article/**Supplementary Material**. Further inquiries can be directed to the corresponding author.
