# Supplementary material for: The bispecific antibody targeting VISTA and PD-L1 shows enhanced tumor inhibitory activity in pancreatic, endometrial and breast cancers compared to mono- and combination immune checkpoint blockade
Source: Front Immunol. 2025 May 9;16:1486799. doi: 10.3389/fimmu.2025.1486799 (PMC12099300; doi:10.3389/fimmu.2025.1486799)
Supplement: Supplementary file 1 [file DataSheet1.docx]

Supplementary Material

Bispecific antibody targeting VISTA and PD-L1 shows enhanced tumor inhibitory activity in pancreatic, endometrial and breast cancers compared to mono- and combination immune checkpoint blockade

Przemysław Bielski^1,2,3^, Jan Barczyński^1,3^, Michał Mikitiuk^1,3^, Maja Myrcha^1^, Kamil Rykała^3^, Louis Boon^3^, Wiktoria Gąsior^3^, Aleksandra Hec-Gałązka^1^, Tad A. Holak^1^, and Tomasz Sitar*^1,3^

^1^Recepton Sp. z o.o., ul. Trzy Lipy 3, 80-172 Gdansk, Poland

^2^Department of Organic Chemistry, Faculty of Chemistry, Jagiellonian University, Gronostajowa 2, 30-387 Krakow, Poland

^3^JJP Biologics Sp. z o.o., ul. Bobrowiecka 6, 00-728 Warsaw, Poland

# Supplementary Figures and Tables

## Supplementary Figures

Table S1. Graphic representation of immune receptors and antibodies constructs.

| **Immune checkpoint** | **ID of UniProt, NIH or Drug bank sequences** | **Construct** |
| --- | --- | --- |
| PD-1 | Q15116 | \| PD-1 ECD (25-167) \| IEGR \| Fc IgG1 \| \| --- \| --- \| --- \| |
| PD-L1 | Q9NZQ7 | \| PD-L1 ECD (19-238) \| HHHHHH \| IEGR \| Fc IgG1 \| \| --- \| --- \| --- \| --- \| |
| VISTA | Q9H7M9 | \| PD-L1 ECD (19-238) \| HHHHHH \| IEGR \| Fc IgG1 \| \| --- \| --- \| --- \| --- \| |
| VSIG-3 | BAC07546.1 | \| VSIG-3 ECD (23-241) \| IEGR \| Fc IgG1 \| \| --- \| --- \| --- \| |
| Ont | 1UI8F5IIZ4 | 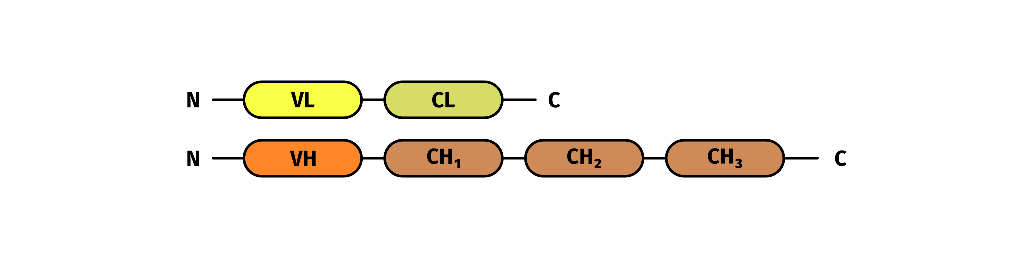 |
| Ate | 52CMI0WC3Y | 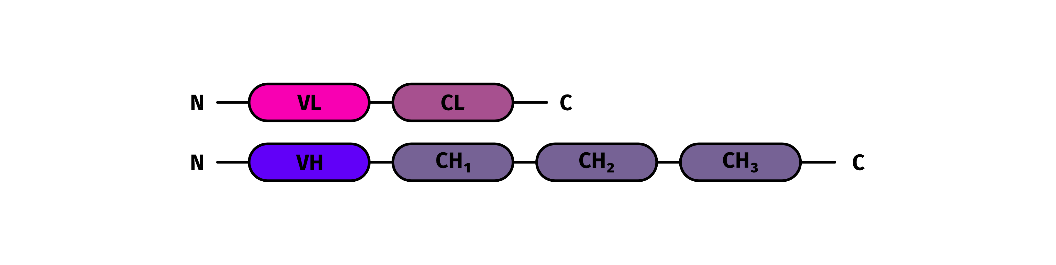 |
| bsAb-1 | N/A | 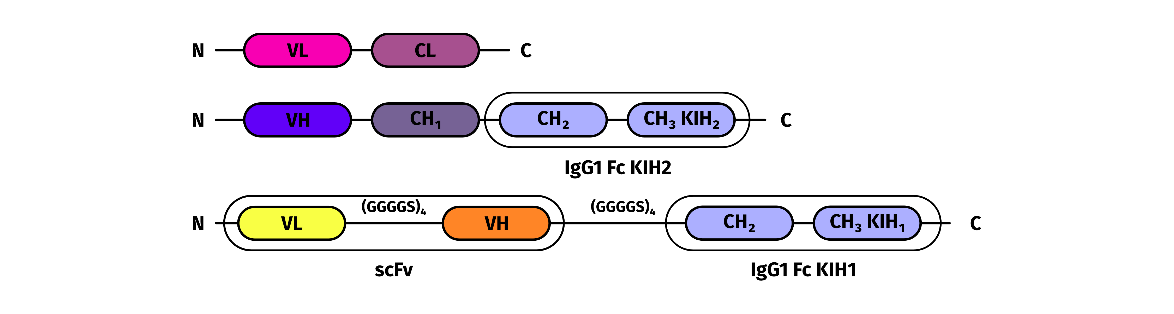 |
| bsAb-2 | N/A | 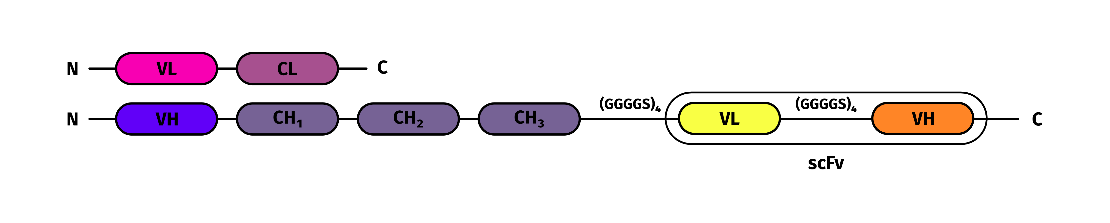 |
| bsAb-3 | N/A | 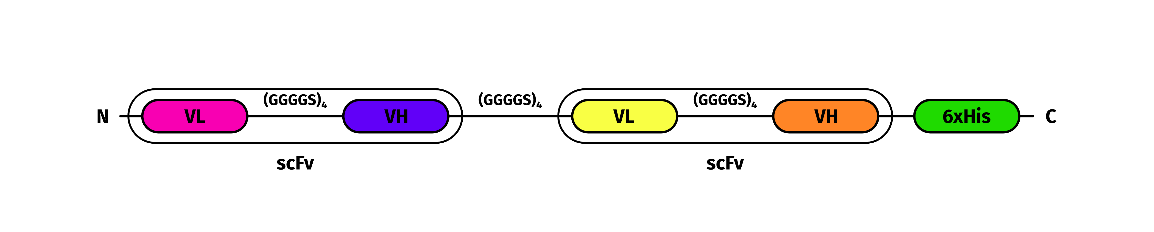 |

**A**

| **Lysis [%]** | | **p value** | | | | | | | |
| --- | --- | --- | --- | --- | --- | --- | --- | --- | --- |
|  |  |  | **bsAb-1** | **bsAb-2** | **bsAb-3** | **Ate** | **Onv** | **Ate+Onv** | **Isotype** |
| **bsAb-1** | **41,41** | **bsAb-1** | **x** | **0,3681** | **0,0022** | **0,0052** | **<0,0001** | **0,0004** | **<0,0001** |
| **bsAb-2** | **49,55** | **bsAb-2** | **0,3681** | **x** | **0,0003** | **0,0006** | **<0,0001** | **<0,0001** | **<0,0001** |
| **bsAb-3** | **16,85** | **bsAb-3** | **0,0022** | **0,0003** | **x** | **0,5771** | **<0,0001** | **0,1223** | **<0,0001** |
| **Ate** | **19,06** | **Ate** | **0,0052** | **0,0006** | **0,5771** | **x** | **0,0001** | **0,0744** | **0,0002** |
| **Onv** | **0,67** | **Onv** | **<0,0001** | **<0,0001** | **<0,0001** | **0,0001** | **x** | **0,0069** | **0,2098** |
| **Ate+Onv** | **10,76** | **Ate+Onv** | **0,0004** | **<0,0001** | **0,1223** | **0,0744** | **0,0069** | **x** | **0,0121** |
| **Isotype** | **1,571** | **Isotype** | **<0,0001** | **<0,0001** | **<0,0001** | **0,0002** | **0,2098** | **0,0121** | **x** |

**B**

| **Lysis [%]** | | **p value** | | | | | | | |
| --- | --- | --- | --- | --- | --- | --- | --- | --- | --- |
|  |  |  | **bsAb-1** | **bsAb-2** | **bsAb-3** | **Ate** | **Onv** | **Ate+Onv** | **Isotype** |
| **bsAb-1** | **55,99** | **bsAb-1** | **x** | **0,3381** | **<0,0001** | **<0,0001** | **<0,0001** | **<0,0001** | **<0,0001** |
| **bsAb-2** | **62,06** | **bsAb-2** | **0,3381** | **x** | **<0,0001** | **<0,0001** | **<0,0001** | **<0,0001** | **<0,0001** |
| **bsAb-3** | **24,67** | **bsAb-3** | **<0,0001** | **<0,0001** | **x** | **0,0434** | **0,001** | **0,0003** | **<0,0001** |
| **Ate** | **15,02** | **Ate** | **<0,0001** | **<0,0001** | **0,0434** | **x** | **0,0942** | **0,0166** | **0,0007** |
| **Onv** | **8,677** | **Onv** | **<0,0001** | **<0,0001** | **0,001** | **0,0942** | **x** | **0,4412** | **0,0188** |
| **Ate+Onv** | **6,376** | **Ate+Onv** | **<0,0001** | **<0,0001** | **0,0003** | **0,0166** | **0,4412** | **x** | **0,0511** |
| **Isotype** | **2,135** | **Isotype** | **<0,0001** | **<0,0001** | **<0,0001** | **0,0007** | **0,0188** | **0,0511** | **x** |

**C**

| **% lysis** | | **p value** | | | | | | | |
| --- | --- | --- | --- | --- | --- | --- | --- | --- | --- |
|  |  |  | **bsAb-1** | **bsAb-2** | **bsAb-3** | **Ate** | **Onv** | **Ate+Onv** | **Isotype** |
| **bsAb-1** | **23,31** | **bsAb-1** | **x** | **0,6938** | **0,0121** | **0,0004** | **0,0002** | **0,0004** | **0,0004** |
| **bsAb-2** | **26,17** | **bsAb-2** | **0,6938** | **x** | **0,0309** | **0,0039** | **0,002** | **0,0038** | **0,004** |
| **bsAb-3** | **10,03** | **bsAb-3** | **0,0121** | **0,0309** | **x** | **0,0279** | **0,0039** | **0,0377** | **0,03** |
| **Ate** | **2,877** | **Ate** | **0,0002** | **0,002** | **0,0279** | **x** | **0,0367** | **0,9638** | **0,9593** |
| **Onv** | **0,081** | **Onv** | **0,0002** | **0,0027** | **0,0039** | **0,0367** | **x** | **0,1648** | **0,037** |
| **Ate+Onv** | **2,79** | **Ate+Onv** | **0,0004** | **0,0038** | **0,0377** | **0,9638** | **0,1648** | **x** | **0,9374** |
| **Isotype** | **2,963** | **Isotype** | **0,0004** | **0,004** | **0,03** | **0,9593** | **0,037** | **0,9374** | **x** |

Figure S1. Antibodies induce PBMCs potential to kill RL95-2, Panc-1 and BT20 cells. The p-values of the lysis values (A,B,C) for RL95-2, BT20, and Panc-1, respectively. Data are derived from human PBMCs from 4 healthy donors. All experiments were repeated three times. Data were considered statistically significant when p values were lower than 0.05 (*p <0.05, **p < 0.01, ***p<0.001 and ****p <0.0001) (Onv: anti-VISTA (Onvatilimab); Ate: anti-PD-L1 (Atezolizumab); bsAb-1: asymmetric bsAb anti-PD-L1/anti-VISTA; bsAb-2: symmetric bsAb anti-PD-L1/anti-VISTA; bsAb-3: 2 x scFv anti-PD-L1/anti-VISTA; Ate+Onv: combination anti-PD-L1+ anti-VISTA; Isotype: Ipilimumab).

**A**

| **con. IFNγ [pg/ml]** | | **p value** | | | | | | | | | |
| --- | --- | --- | --- | --- | --- | --- | --- | --- | --- | --- | --- |
|  |  |  | **bsAb-1** | **bsAb-2** | **bsAb-3** | **Ate** | **Onv** | **Ate+Onv** | **RL95-2/PBMC** | **PBMC** | **Isotype** |
| **bsAb-1** | **596.2** | **bsAb-1** | **x** | **<0,0001** | **0.0006** | **0.0002** | **0.0002** | **<0,0001** | **<0,0001** | **<0,0001** | **<0,0001** |
| **bsAb-2** | **2337** | **bsAb-2** | **<0,0001** | **x** | **<0,0001** | **<0,0001** | **<0,0001** | **<0,0001** | **<0,0001** | **<0,0001** | **<0,0001** |
| **bsAb-3** | **847.8** | **bsAb-3** | **0.0006** | **<0,0001** | **x** | **<0,0001** | **<0,0001** | **<0,0001** | **<0,0001** | **<0,0001** | **<0,0001** |
| **Ate** | **352.4** | **Ate** | **0.0002** | **<0,0001** | **<0,0001** | **x** | **0.023** | **0.5338** | **0.3463** | **0.0077** | **0.0239** |
| **Onv** | **199.5** | **Onv** | **<0,0001** | **<0,0001** | **<0,0001** | **0.023** | **x** | **0.6344** | **0.8242** | **0.9997** | **>0,9999** |
| **Ate+Onv** | **272.7** | **Ate+Onv** | **<0,0001** | **<0,0001** | **<0,0001** | **0.5338** | **0.6344** | **x** | **>0,9999** | **0.3287** | **0.6466** |
| **RL95-2/PBMC** | **259.3** | **RL95-2/PBMC** | **<0,0001** | **<0,0001** | **<0,0001** | **0.3463** | **0.8242** | **>0,9999** | **x** | **0.512** | **0.8338** |
| **PBMC** | **178.1** | **PBMC** | **<0,0001** | **<0,0001** | **<0,0001** | **0.0077** | **0.9997** | **0.3287** | **0.512** | **x** | **0.9996** |
| **Isotype** | **200.3** | **Isotype** | **<0,0001** | **<0,0001** | **<0,0001** | **0.0239** | **>0,9999** | **0.6466** | **0.8338** | **0.9996** | **x** |

**B**

| **con. TNFα [pg/ml]** | | **p value** | | | | | | | | | |
| --- | --- | --- | --- | --- | --- | --- | --- | --- | --- | --- | --- |
|  |  |  | **bsAb-1** | **bsAb-2** | **bsAb-3** | **Ate** | **Onv** | **Ate+Onv** | **RL95-2/PBMC** | **PBMC** | **Isotype** |
| **bsAb-1** | **521** | **bsAb-1** | **x** | **<0,0001** | **<0,0001** | **<0,0001** | **<0,0001** | **<0,0001** | **<0,0001** | **<0,0001** | **<0,0001** |
| **bsAb-2** | **828.7** | **bsAb-2** | **<0,0001** | **x** | **<0,0001** | **<0,0001** | **<0,0001** | **<0,0001** | **<0,0001** | **0.0003** | **<0,0001** |
| **bsAb-3** | **349.9** | **bsAb-3** | **<0,0001** | **<0,0001** | **x** | **0.781** | **0.0003** | **0.4454** | **0.2576** | **<0,0001** | **0.0119** |
| **Ate** | **318.6** | **Ate** | **<0,0001** | **<0,0001** | **0.781** | **x** | **0.0093** | **0.9995** | **0.9849** | **<0,0001** | **0.2559** |
| **Onv** | **235.1** | **Onv** | **<0,0001** | **<0,0001** | **0.0003** | **0.0093** | **x** | **0.0302** | **0.0646** | **<0,0001** | **0.7143** |
| **Ate+Onv** | **307.5** | **Ate+Onv** | **<0,0001** | **<0,0001** | **0.4454** | **0.9995** | **0.0302** | **x** | **>0,9999** | **<0,0001** | **0.5528** |
| **RL95-2/PBMC** | **300.1** | **RL95-2/PBMC** | **<0,0001** | **<0,0001** | **0.2576** | **0.9849** | **0.0646** | **>0,9999** | **x** | **<0,0001** | **0.7778** |
| **PBMC** | **945** | **PBMC** | **<0,0001** | **0.0003** | **<0,0001** | **<0,0001** | **<0,0001** | **<0,0001** | **<0,0001** | **x** | **<0,0001** |
| **Isotype** | **268.7** | **Isotype** | **<0,0001** | **<0,0001** | **0.0119** | **0.2559** | **0.7143** | **0.5528** | **0.7778** | **<0,0001** | **x** |

**C**

| **con. Granzyme B [pg/ml]** | | **p value** | | | | | | | | | | |
| --- | --- | --- | --- | --- | --- | --- | --- | --- | --- | --- | --- | --- |
|  |  |  | **bsAb-1** | **bsAb-2** | **bsAb-3** | **Ate** | **Onv** | **Ate+Onv** | | **RL95-2/PBMC** | **PBMC** | **Isotype** |
| **bsAb-1** | **1650** | **bsAb-1** | **x** | **<0,0001** | **<0,0001** | **<0,0001** | **<0,0001** | **<0,0001** | **<0,0001** | | **<0,0001** | **<0,0001** |
| **bsAb-2** | **2807** | **bsAb-2** | **<0,0001** | **x** | **<0,0001** | **<0,0001** | **<0,0001** | **<0,0001** | **<0,0001** | | **<0,0001** | **<0,0001** |
| **bsAb-3** | **486.4** | **bsAb-3** | **<0,0001** | **<0,0001** | **x** | **0.8833** | **0.1295** | **0.9728** | **0.4071** | | **0.0034** | **0.1936** |
| **Ate** | **622.7** | **Ate** | **<0,0001** | **<0,0001** | **0.8833** | **x** | **0.7957** | **>0,9999** | **0.9922** | | **0.0593** | **0.898** |
| **Onv** | **331.2** | **Onv** | **<0,0001** | **<0,0001** | **0.1295** | **0.7957** | **x** | **0.6017** | **0.9976** | | **0.6697** | **>0,9999** |
| **Ate+Onv** | **519.3** | **Ate+Onv** | **<0,0001** | **<0,0001** | **0.9728** | **>0,9999** | **0.6017** | **x** | **0.9948** | | **0.0304** | **0.739** |
| **RL95-2/PBMC** | **402** | **RL95-2/PBMC** | **<0,0001** | **<0,0001** | **0.4071** | **0.9922** | **0.9976** | **0.9948** | **x** | | **0.2732** | **0.9998** |
| **PBMC** | **154.1** | **PBMC** | **<0,0001** | **<0,0001** | **0.0034** | **0.0593** | **0.6697** | **0.0304** | **0.2732** | | **x** | **0.5296** |
| **Isotype** | **353.9** | **Isotype** | **<0,0001** | **<0,0001** | **0.1936** | **0.898** | **>0,9999** | **0.739** | **0.9998** | | **0.5296** | **x** |

**D**

| **con. IL-2 [pg/ml]** | | **p value** | | | | | | | | | |
| --- | --- | --- | --- | --- | --- | --- | --- | --- | --- | --- | --- |
|  |  |  | **bsAb-1** | **bsAb-2** | **bsAb-3** | **Ate** | **Onv** | **Ate+Onv** | **RL95-2/PBMC** | **PBMC** | **Isotype** |
| **bsAb-1** | **0** | **bsAb-1** | **x** | **N/A** | **<0,0001** | **<0,0001** | **N/A** | **N/A** | **N/A** | **N/A** | **N/A** |
| **bsAb-2** | **0** | **bsAb-2** | **N/A** | **x** | **<0,0001** | **<0,0001** | **N/A** | **N/A** | **N/A** | **N/A** | **N/A** |
| **bsAb-3** | **48.16** | **bsAb-3** | **<0,0001** | **<0,0001** | **x** | **0.1683** | **<0,0001** | **<0,0001** | **<0,0001** | **<0,0001** | **<0,0001** |
| **Ate** | **89.61** | **Ate** | **<0,0001** | **<0,0001** | **0.1683** | **x** | **<0,0001** | **<0,0001** | **<0,0001** | **<0,0001** | **<0,0001** |
| **Onv** | **0** | **Onv** | **N/A** | **N/A** | **<0,0001** | **<0,0001** | **x** | **N/A** | **N/A** | **N/A** | **N/A** |
| **Ate+Onv** | **0** | **Ate+Onv** | **N/A** | **N/A** | **<0,0001** | **<0,0001** | **N/A** | **x** | **N/A** | **N/A** | **N/A** |
| **RL95-2/PBMC** | **0** | **RL95-2/PBMC** | **N/A** | **N/A** | **<0,0001** | **<0,0001** | **N/A** | **N/A** | **x** | **N/A** | **N/A** |
| **PBMC** | **0** | **PBMC** | **N/A** | **N/A** | **<0,0001** | **<0,0001** | **N/A** | **N/A** | **N/A** | **x** | **N/A** |
| **Isotype** | **0** | **Isotype** | **N/A** | **N/A** | **<0,0001** | **<0,0001** | **N/A** | **N/A** | **N/A** | **N/A** | **x** |

**E**

| **con. IL-10 [pg/ml]** | | **p value** | | | | | | | | | |
| --- | --- | --- | --- | --- | --- | --- | --- | --- | --- | --- | --- |
|  |  |  | **bsAb-1** | **bsAb-2** | **bsAb-3** | **Ate** | **Onv** | **Ate+Onv** | **RL95-2/PBMC** | **PBMC** | **Isotype** |
| **bsAb-1** | **281.2** | **bsAb-1** | **x** | **0.0148** | **<0,0001** | **<0,0001** | **<0,0001** | **<0,0001** | **<0,0001** | **<0,0001** | **<0,0001** |
| **bsAb-2** | **455** | **bsAb-2** | **0.0148** | **x** | **<0,0001** | **<0,0001** | **<0,0001** | **<0,0001** | **<0,0001** | **<0,0001** | **<0,0001** |
| **bsAb-3** | **0** | **bsAb-3** | **<0,0001** | **<0,0001** | **x** | **N/A** | **N/A** | **N/A** | **N/A** | **N/A** | **N/A** |
| **Ate** | **0** | **Ate** | **<0,0001** | **<0,0001** | **N/A** | **x** | **N/A** | **N/A** | **N/A** | **N/A** | **N/A** |
| **Onv** | **0** | **Onv** | **<0,0001** | **<0,0001** | **N/A** | **N/A** | **x** | **N/A** | **N/A** | **N/A** | **N/A** |
| **Ate+Onv** | **0** | **Ate+Onv** | **<0,0001** | **<0,0001** | **N/A** | **N/A** | **N/A** | **x** | **N/A** | **N/A** | **N/A** |
| **RL95-2/PBMC** | **0** | **RL95-2/PBMC** | **<0,0001** | **<0,0001** | **N/A** | **N/A** | **N/A** | **N/A** | **x** | **N/A** | **N/A** |
| **PBMC** | **0** | **PBMC** | **<0,0001** | **<0,0001** | **N/A** | **N/A** | **N/A** | **N/A** | **N/A** | **x** | **N/A** |
| **Isotype** | **0** | **Isotype** | **<0,0001** | **<0,0001** | **N/A** | **N/A** | **N/A** | **N/A** | **N/A** | **N/A** | **x** |

Figure S2. Antibodies induce cytokine release by PBMCs co-cultured with RL95.

The p-values for the INF-γ, TNFα, Granzyme B, IL-2 and IL-10 concentration (A, B, C, D, E) respectively. Data are derived from human PBMCs from 1 healthy donor. All experiments were repeated three times. Data was considered statistically significant when p values lower than 0.05 (*p <0.05, **p < 0.01, ***p<0.001 and ****p <0.0001) (Onv: anti-VISTA (Onvatilimab); Ate: anti-PD-L1 (Atezolizumab); bsAb-1: asymmetric bsAb anti-PD-L1/anti-VISTA; bsAb-2: symmetric bsAb anti-PD-L1/anti-VISTA; bsAb-3: 2 x scFv anti-PD-L1/anti-VISTA; Ate+Onv: combination anti-PD-L1+ anti-VISTA; RL95-2/PBMC: coculture RL95-2 with PBMC without antibodies; PBMC: PBMC alone; Isotype: Ipilimumab).

**A**

| **CD8+ [%]** | | **p value** | | | | | | |
| --- | --- | --- | --- | --- | --- | --- | --- | --- |
|  |  |  | **bsAb-1** | **bsAb-2** | **Ate** | **Onv** | **Ate+Onv** | **PBMC alone** |
| **bsAb-1** | **22,23** | **bsAb-1** | **x** | **0.9995** | **0.4382** | **0.503** | **0.1417** | **0.0135** |
| **bsAb-2** | **22,10** | **bsAb-2** | **0.9995** | **x** | **0.6125** | **0.6816** | **0.2279** | **0.0229** |
| **Ate** | **21,43** | **Ate** | **0.4382** | **0.6125** | **x** | **>0,9999** | **0.9612** | **0.2961** |
| **Onv** | **21,48** | **Onv** | **0.503** | **0.6816** | **>0,9999** | **x** | **0.9322** | **0.25** |
| **Ate+Onv** | **21,10** | **Ate+Onv** | **0.1417** | **0.2279** | **0.9612** | **0.9322** | **x** | **0.7177** |
| **PBMC alone** | **20,51** | **PBMC alone** | **0.0135** | **0.0229** | **0.2961** | **0.25** | **0.7177** | **x** |

**B**

| **CD4+ [%]** | | **p value** | | | | | | |
| --- | --- | --- | --- | --- | --- | --- | --- | --- |
|  |  |  | **bsAb-1** | **bsAb-2** | **Ate** | **Onv** | **Ate+Onv** | **PBMC alone** |
| **bsAb-1** | **53.28** | **bsAb-1** | **x** | **0.681** | **0.0002** | **0.4894** | **0.0002** | **<0,0001** |
| **bsAb-2** | **53.74** | **bsAb-2** | **0.681** | **x** | **<0,0001** | **0.055** | **<0,0001** | **<0,0001** |
| **Ate** | **50.35** | **Ate** | **0.0002** | **<0,0001** | **x** | **0.0025** | **>0,9999** | **0.8992** |
| **Onv** | **51.91** | **Onv** | **0.4894** | **0.055** | **0.0025** | **x** | **0.0034** | **0.0005** |
| **Ate+Onv** | **50.93** | **Ate+Onv** | **0.0002** | **<0,0001** | **>0,9999** | **0.0034** | **x** | **0.8162** |
| **PBMC alone** | **49.66** | **PBMC alone** | **<0,0001** | **<0,0001** | **0.8992** | **0.0005** | **0.8162** | **x** |

**C**

| **CD25+FoxP3+ [%]** | | **p value** | | | | | | |
| --- | --- | --- | --- | --- | --- | --- | --- | --- |
|  |  |  | **bsAb-1** | **bsAb-2** | **Ate** | **Onv** | **Ate+Onv** | **PBMC alone** |
| **bsAb-1** | **4.80** | **bsAb-1** | **x** | **0.1087** | **0.0022** | **0.0044** | **0.0079** | **0.0061** |
| **bsAb-2** | **4.62** | **bsAb-2** | **0.1087** | **x** | **0.2453** | **0.4334** | **0.6305** | **0.5436** |
| **Ate** | **3.55** | **Ate** | **0.0022** | **0.2453** | **x** | **0.9976** | **0.9649** | **0.986** |
| **Onv** | **3.49** | **Onv** | **0.0044** | **0.4334** | **0.9976** | **x** | **0.9991** | **>0,9999** |
| **Ate+Onv** | **3.60** | **Ate+Onv** | **0.0079** | **0.6305** | **0.9649** | **0.9991** | **x** | **>0,9999** |
| **PBMC alone** | **3.39** | **PBMC alone** | **0.0061** | **0.5436** | **0.986** | **>0,9999** | **>0,9999** | **x** |

**E**

| **M_MDSC** | |  | | | | | | |
| --- | --- | --- | --- | --- | --- | --- | --- | --- |
|  |  |  | **bsAb-1** | **bsAb-2** | **Ate** | **Onv** | **Ate+Onv** | **PBMC alone** |
| **bsAb-1** | **1.34** | **bsAb-1** | **x** | **0.8725** | **0.0016** | **0.0009** | **0.0009** | **0.0011** |
| **bsAb-2** | **1.10** | **bsAb-2** | **0.8725** | **x** | **0.0094** | **0.0049** | **0.0054** | **0.0066** |
| **Ate** | **0.14** | **Ate** | **0.0016** | **0.0094** | **x** | **0.9985** | **0.9993** | **>0,9999** |
| **Onv** | **0.05** | **Onv** | **0.0009** | **0.0049** | **0.9985** | **x** | **>0,9999** | **>0,9999** |
| **Ate+Onv** | **0.06** | **Ate+Onv** | **0.0009** | **0.0054** | **0.9993** | **>0,9999** | **x** | **>0,9999** |
| **PBMC alone** | **0.09** | **PBMC alone** | **0.0011** | **0.0066** | **>0,9999** | **>0,9999** | **>0,9999** | **x** |

**F**

| **PMN-MDSC** | | **p value** | | | | | | |
| --- | --- | --- | --- | --- | --- | --- | --- | --- |
|  |  |  | **bsAb-1** | **bsAb-2** | **Ate** | **Onv** | **Ate+Onv** | **PBMC alone** |
| **bsAb-1** | **8.52** | **bsAb-1** | **x** | **0.9342** | **0.0011** | **0.0005** | **0.0007** | **0.0656** |
| **bsAb-2** | **7.33** | **bsAb-2** | **0.9342** | **x** | **0.005** | **0.0022** | **0.003** | **0.274** |
| **Ate** | **1.19** | **Ate** | **0.0011** | **0.005** | **x** | **0.9948** | **0.9996** | **0.2106** |
| **Onv** | **0.53** | **Onv** | **0.0005** | **0.0022** | **0.9948** | **x** | **>0,9999** | **0.0952** |
| **Ate+Onv** | **0.80** | **Ate+Onv** | **0.0007** | **0.003** | **0.9996** | **>0,9999** | **x** | **0.1323** |
| **PBMC alone** | **4.38** | **PBMC alone** | **0.0656** | **0.274** | **0.2106** | **0.0952** | **0.1323** | **x** |

Figure S3. Leukocytes phenotyping and killing effect of PBMCs on tumor cells assay.

The p-values for the CD4+, CD8+, CD25+FoxP+, CD14+HLA-DR- and CD15+HLA-DR- level (A, B, C, D, E, F) respectively. Gating path: CD4+ (CD45+>CD3+>CD4+), CD8+ (CD45+>CD3+>CD8+), CD25+FoxP+ (CD45+>CD3+>CD4+>CD25+>FoxP+), CD14+HLA-DR- (CD45+>CD11b+>CD14+>HLA-DR-), CD15+HLA-DR- (CD45+> CD11b+> CD15+>HLA-DR-). Data are derived from human PBMCs from 1 healthy donor. All experiments for leukocytes phenotyping were repeated three times. Data was considered statistically significant when p values lower than 0.05 (*p <0.05, **p < 0.01, ***p<0.001 and ****p <0.0001) (Onv: anti-VISTA (Onvatilimab); Ate: anti-PD-L1 (Atezolizumab); bsAb-1: asymmetric bsAb anti-PD-L1/anti-VISTA; bsAb-2: symmetric bsAb anti-PD-L1/anti-VISTA; Ate+Onv: combination anti-PD-L1+ anti-VISTA; PBMC alone: PBMC).
